# Correlation between age, sex, and severity of Coronavirus disease-19 based on chest computed tomography severity scoring system

**DOI:** 10.1186/s43055-021-00408-1

**Published:** 2021-01-13

**Authors:** Shimaa Farghaly, Marwa Makboul

**Affiliations:** Radio-Diagnosis Department, Faculty of Medicine, Assiut University, Assiut, Egypt

**Keywords:** Coronavirus disease, Computed tomography, Computed tomography severity score, Coronavirus disease imaging reporting system

## Abstract

**Background:**

Coronavirus disease 2019 (COVID-19) is the most recent global health emergency; early diagnosis of COVID-19 is very important for rapid clinical interventions and patient isolation; chest computed tomography (CT) plays an important role in screening, diagnosis, and evaluating the progress of the disease. According to the results of different studies, due to high severity of the disease, clinicians should be aware of the different potential risk factors associated with the fatal outcome, so chest CT severity scoring system was designed for semi-quantitative assessment of the severity of lung disease in COVID-19 patients, ranking the pulmonary involvement on 25 points severity scale according to extent of lung abnormalities; this study aims to evaluate retrospectively the relationship between age and severity of COVID-19 in both sexes based on chest CT severity scoring system.

**Results:**

Age group C (40–49 year) was the commonest age group that was affected by COVID-19 by 21.3%, while the least affected group was group F (≥ 70 years) by only 6.4%. As regards COVID-RADS classification, COVID-RADS-3 was the most commonly presented at both sexes in all different age groups. Total CT severity lung score had a positive strong significant correlation with the age of the patient (*r =* 0.64, *P* < 0.001). Also, a positive strong significant correlation was observed between CT severity lung score and age in both males and females (*r =* 0.59, *P* < 0.001) and (*r =* 0.69, *P* < 0.001) respectively.

**Conclusion:**

We concluded that age can be considered as a significant risk factor for the severity of COVID-19 in both sexes. Also, CT can be used as a significant diagnostic tool for the diagnosis of COVID-19 and evaluation of the progression and severity of the disease.

## Background

Coronavirus disease 2019 (COVID-19) is the most recent global health emergency, which is first developed at the end of 2019 in China, (Wuhan). It is caused by a beta-coronavirus called coronavirus 2 (SARS-CoV) [1, 2].

Early diagnosis of COVID-19 is very important for rapid clinical interventions and patient isolation [3]. Fever, dry cough, sore throat, and fatigue are the main clinical manifestations of COVID-19. However, other symptoms may develop such as headache, nasal obstruction, myalgia, loss of smell and taste, and diarrhea. Also, acute respiratory distress syndrome (ARDS) may rapidly develop in seriously ill patients [4, 5].

A specific viral test using real reverse transcription-polymerase chain reaction (RT-PCR) was quickly developed to confirm the diagnosis of COVID-19. Although it is considered the gold standard test for diagnosis, however, some patients might have false-negative results [5]. This may be due to insufficient cellular material for detection and improper extraction of nucleic acid from clinical materials [6, 7]. The variable results of RT-PCR emphasize the need for an additional diagnostic approach [3, 6].

Chest computed tomography (CT) plays an important role in screening, diagnosing, and evaluating the progress of the disease according to the results of different studies. Also, it may manifest abnormalities earlier than RT-PCR testing and yields a typical pattern with 97% sensitivity [8, 9]. So, the Coronavirus disease imaging reporting system (COVID-RADS) classification was developed based on CT findings aiming to standardize CT report in different COVID-19 patients, which will be helpful in clinical diagnosis and research applications [10].

Ground-glass opacification, consolidation, linear opacities, and crazy-paving pattern are the predominant patterns of COVID-19 on CT scan. However, other minor signs such as air bronchogram, pulmonary nodules, cavitation, pleural and pericardial effusion, bronchiectasis, pneumothorax, and mediastinal lymphadenopathy were also noted [11].

Due to the high severity of the disease, clinicians should be aware of the different potential risk factors associated with fatal outcome [12, 13], so a chest CT severity scoring system was designed for semi-quantitative assessment of the severity of lung disease in COVID-19 patients, ranking the pulmonary involvement on 25 points severity scale according to extent of lung abnormalities [14].

Regarding the intimate relationship between the risk of death in COVID-19 patients and pulmonary involvement [15], so the study aimed to evaluate retrospectively the relationship between age and severity of COVID-19 in both sexes based on chest CT severity scoring system.

## Methods

This retrospective study was conducted on 574 patients who were diagnosed as COVID-19 and confirmed by real-time polymerase chain reaction (RT-PCR) during the period from 1 April 2020 to 1 July 2020, and no exclusion criteria were applied.

These selected patients were classified according to their age into six groups: (group A) less than 30 years, (B) from 31 to 39 years, (C) from 40 to 49 years, (D) 50–59 years, (E) from 60 to 69 years, and (F) 70 years and more.

The study was carried out after obtaining the permission of the Ethics Committee of Scientific Research, Faculty of Medicine, and as the study had no risk and does not affect the patients’ rights, the informed consent was waived.

All patients underwent non-contrast multi-slice computed tomography (MSCT) chest for evaluating the severity of the disease in different age groups.

### CT protocol

All patients underwent chest CT examination without intravenous contrast in a supine position; CT images were acquired in the caudo-cranial direction from the level of diaphragm to lung apices, using 64-channel multi-detector CT scanner (Toshiba, Japan) Aquilion machine with 120–140 kV, 16 × 1.2 mm collimation, and tube current 150–280 mA; all transverse images were reconstructed to 0.625 mm-slice images.

### CT chest image interpretation

Images were transferred to a Vitrea Vital Image (VPMC-Revision C) and multi-planar reconstruction (MPR) was used for image analysis.

CT images were reviewed by two radiologists with more than 10 years’ experience in imaging.

For each patient, a CT scan was evaluated for the following:

1. Lesion density: ground glass, mixed ground glass with consolidation, or only consolidation.
2. Lesion distribution: peripheral (sub-pleural, involve peripheral one-third of the lung), central (at lung hilum, involve central two-thirds of the lung), or diffuse.
3. Other signs: such as air bronchogram, pulmonary nodules, cavitation, pleural or pericardial effusion, fibrotic bands, bronchiectasis, or adenopathy.
4. The number of lobes affected.
5. Unilateral or bilateral involvement.

Coronavirus disease imaging reporting system (COVID-RADS) classification was also applied for each patient based on CT findings [10]. This is summarized in Table 1.

**Table 1:** COVID-19 imaging reporting and data system classification

COVID-Rads Grade	CT findings	Description	Level of suspicion
0	Normal CT chest	No findings	Low
1	Atypical findings being (inconsistent with COVID-19).	Pleural effusion, cavity, lymphadenopathy, halo sign, a tree in bud sign, bronchiectasis, pulmonary emphysema, pulmonary fibrosis, pneumothorax, or pericardial effusion.	Low
2A	Fairly typical findings.	Single GGO, focal pleural thickening, consolidation without GGO, vascular enlargement, air bronchogram, bronchial wall thickening, white lung stage, or parenchymal fibrotic bands.	Moderate
2B	Combination of atypical findings with typical/ fairly typical findings.		Moderate
3	Typical findings	Multifocal GGO, GGO with superimposed consolidation, consolidation predominant, crazy paving appearance, linear opacities, or melted sugar sign.	High

Also, the CT severity score was calculated by division of both lungs into five lung zones (right upper lobe, right middle lobe, right lower lobe, left upper lobe (including lingula), and left lower lobe) as regard anatomical structures.

A score was given for each lung lobe based on the percentage of lobe involvement:

- Score 0: 0% involvement
- Score 1: less than 5% involvement
- Score 2: 5% to less than 25% involvement
- Score 3: 25% to less than 50% involvement
- Score 4: 50% to less than 75% involvement
- Score 5: 75% or greater involvement

The maximum CT score for both lungs is 25, and the summation of both lung scores provides a semi-quantitative evaluation of the total severity score.

Then, according to the percentage of whole lung involvement, the grade of disease severity was classified into:

- None: 0%
- Minimal: 1–25%
- Mild: 26-50%
- Moderate: 51–75%
- Sever: 76–100%

Finally, the total severity score and grading were calculated for each patient, and correlation with age in both sexes was done.

### Statistical analysis

Data was collected and analyzed using SPSS (Statistical Package for the Social Science, version 20, IBM, and Armonk, New York). Continuous data were expressed in form of mean ± SD or median (range) while nominal data were expressed in form of frequency (percentage).

The normality test for the age and total lung score was performed by the Shapiro test where it was significant. Hence, age and lung scores were not normally distributed data. *Chi*^*2*^ test was used to compare nominal data in the study while continuous data were compared either by Mann–Whitney *U* test (in case of two different groups) or Kruskal–Wallis test (in case of more than two different groups).

Spearman rank correlation was used to determine correlation between age and total lung score (mild correlation if *r =* 0.20–0.40, moderate correlation if *r =* 0.40–0.60, strong correlation if *r =* 0.60–0.90, and perfect correlation if *r =* 1). Level of confidence was kept at 95% and hence, *P* value was considered significant if < 0.05.

## Results

Five hundred seventy-four patients (311 males and 263 females) with mean age 46.70 ± 15.18 years and age range between 1 and 85 years who confirmed to have COVID-19 were enrolled in this study.

In the current study, we found that age group C (40–49 year) was the commonest age group that affected by COVID-19 by 122 (21.3%) patients, followed by group D (50–59 year) by 118 (20.6%) patients, while the least affected group was group F (≥ 70 years) by only 37 (6.4%) patients.

No lung affection was observed at chest CT in 47 (8.2%) patients, while 461 (80.3%) patients had bilateral lung affection and only 66 (11.5%) patients had unilateral affection.

COVID-RADS-3 was presented in 442 (77%) patients, followed by COVID-RADS-0 in 47 (8.2%) patients, while COVID-RADS-2A and COVID-RADS-2B were presented in 39 (6.8%) and 46 (8%) patients respectively.

Peripheral and diffuse lung involvement was noticed in 314 (54.7%) and 209 (36.4%) patients respectively, while central lung involvement was only noticed in 4 (0.70%) patients. The mean total lung involvement CT score was 10.09 ± 7.06 with the percentage of involvement was 57.63 ± 4.16%.

Of the included patients, 156 (24.7%), 159 (27.7%), 136 (23.7%), and 76 (13.2%) patients had minimal, mild, moderate, and severe grades of affection respectively based on CT severity score. All these results are summarized in Table 2.

**Table 2:** Characteristics of the study population

	*N* = 574
Age (years)	46.70 ± 15.18
Range	1–85
Age groups	
(A) < 30 year	81 (14.1%)
(B) 31– year	115 (20%)
(C) 40–49 year	122 (21.3%)
(D) 50–59 year	118 (20.6%)
(E) 60–69 year	101 (17.6%)
(F) ≥ 70 year	37 (6.4%)
Sex	
Male	311 (54.2%)
Female	263 (45.8%)
CO-RADS	
0	47 (8.2%)
2A	39 (6.8%)
2B	46 (8%)
3	442 (77%)
Laterality of involvement	
None	47 (8.2%)
Unilateral	66 (11.5%)
Bilateral	461 (80.3%)
Distribution of lesions	
None	47 (8.2%)
Peripheral	314 (54.7%)
Central	4 (0.70%)
Diffuse	209 (36.4%)
Lung involvement score	
Right lung score	5.96 ± 4.13
Left lung score	4.13 ± 2.92
Total score	10.09 ± 7.06
Percentage	57.63 ± 4.16
Grade of affection	
None	47 (8.2%)
Minimal	156 (24.7%)
Mild	159 (27.7%)
Moderate	136 (23.7%)
Severe	76 (13.2%)

Data expressed as mean (SD), frequency (percentage)

As regards COVID-RADS classification, we found that COVID-RADS-3 with typical chest CT findings was the most commonly presented at both sexes in all different age groups (Table 3).

**Table 3:** COVID-RADS among the study population based on sex and age group

Sex-based on age groups	CO-RADS			
	0	2A	2B	3
< 30 year				
Male (*n* = 42 (51.9)	13 (31)	9 (21.4)	1 (2.4)	19 (45.2)
Female (*n* = 39 (48.1)	13 (33.3)	10 (25.6)	0	16 (41)
31–39 year				
Male (*n* = 53 (46.1)	4 (7.5)	5 (9.4)	6 (11.3)	38 (71.7)
Female (*n* = 62 (53.9 )	8 (12.9)	4 (6.5)	2 (3.2)	48 (77.4)
40–49 year				
Male (*n* = 72 (59)	2 (2.8)	4 (5.6)	7 (9.7)	59 (81.9)
Female (*n* = 50 (41)	2 (4)	1 (2)	3 (6)	44 (88)
50–59 year				
Male (*n* = 66 (55.9)	1 (1.5)	2 (3)	6 (9.1)	57 (86.4)
Female (*n* = 52 (44.1)	3 (5.8)	3 (5.8)	5 (9.6)	41 (78.8)
60–69 year				
Male (*n* = 58 (57.4)	1 (1.7)	0	5 (8.6)	52 (89.7)
Female (*n* = 43 (42.6)	0	0	4 (9.3)	39 (90.7)
≥ 70 year				
Male (*n* = 20 (54.1)	0	1 (5)	5 (25)	14 (70)
Female (*n* = 17 (45.9)	0	0	2 (11.8)	15 (88.2)

Data expressed as frequency (percentage)

While regarding the relationship between age, sex, and severity of COVID-19, we found that there was a highly statistically significant difference between age and total CT severity lung score with (*P* value < 0.001), where age group A patients (< 30 year old) had the lowest total CT lung score, while the highest score was observed in group F patients (≥ 70 years). Also, in both sexes total, CT severity lung score was increasing with age.

**Fig. 1:**
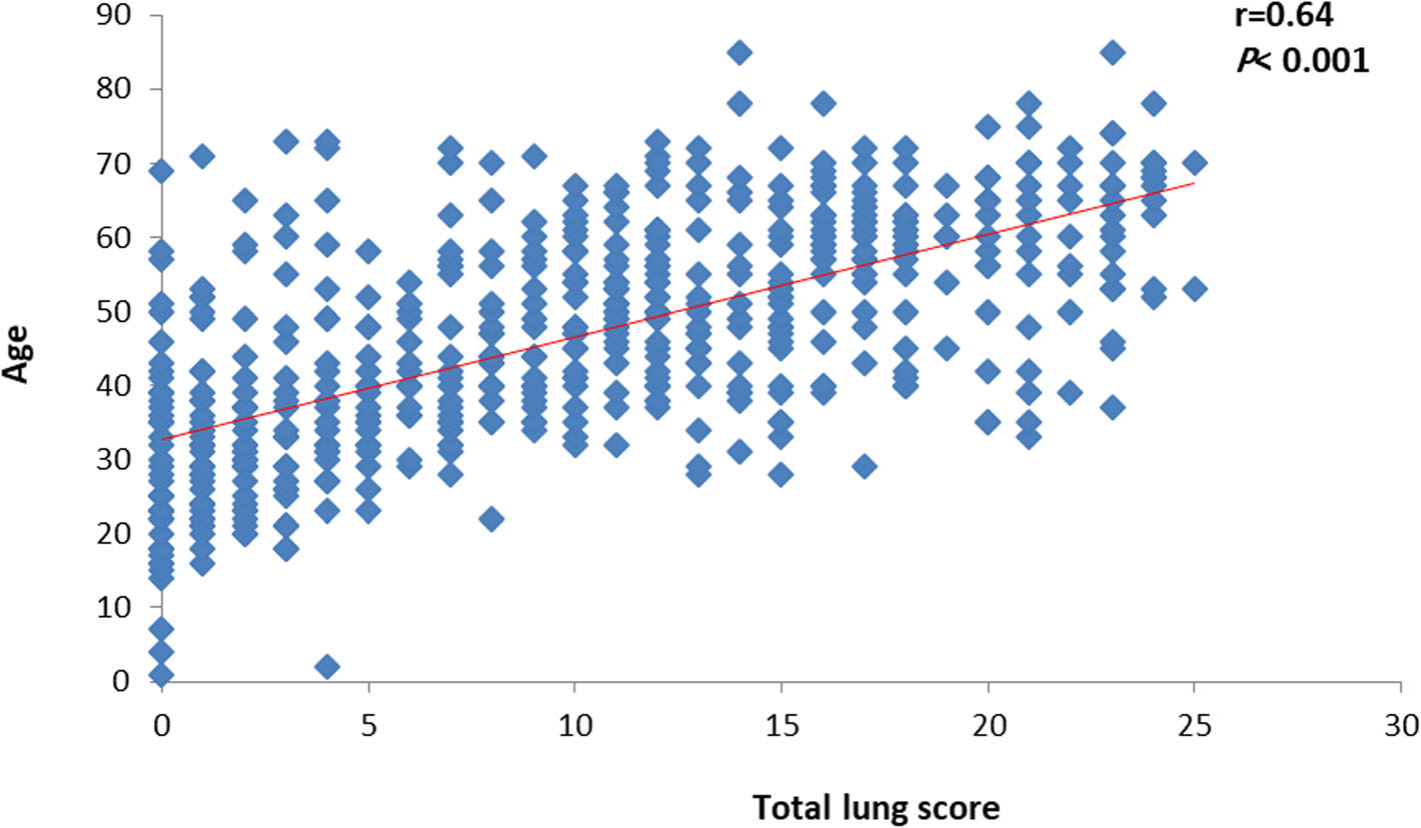
Correlation between total lung score and age

**Fig. 2:**
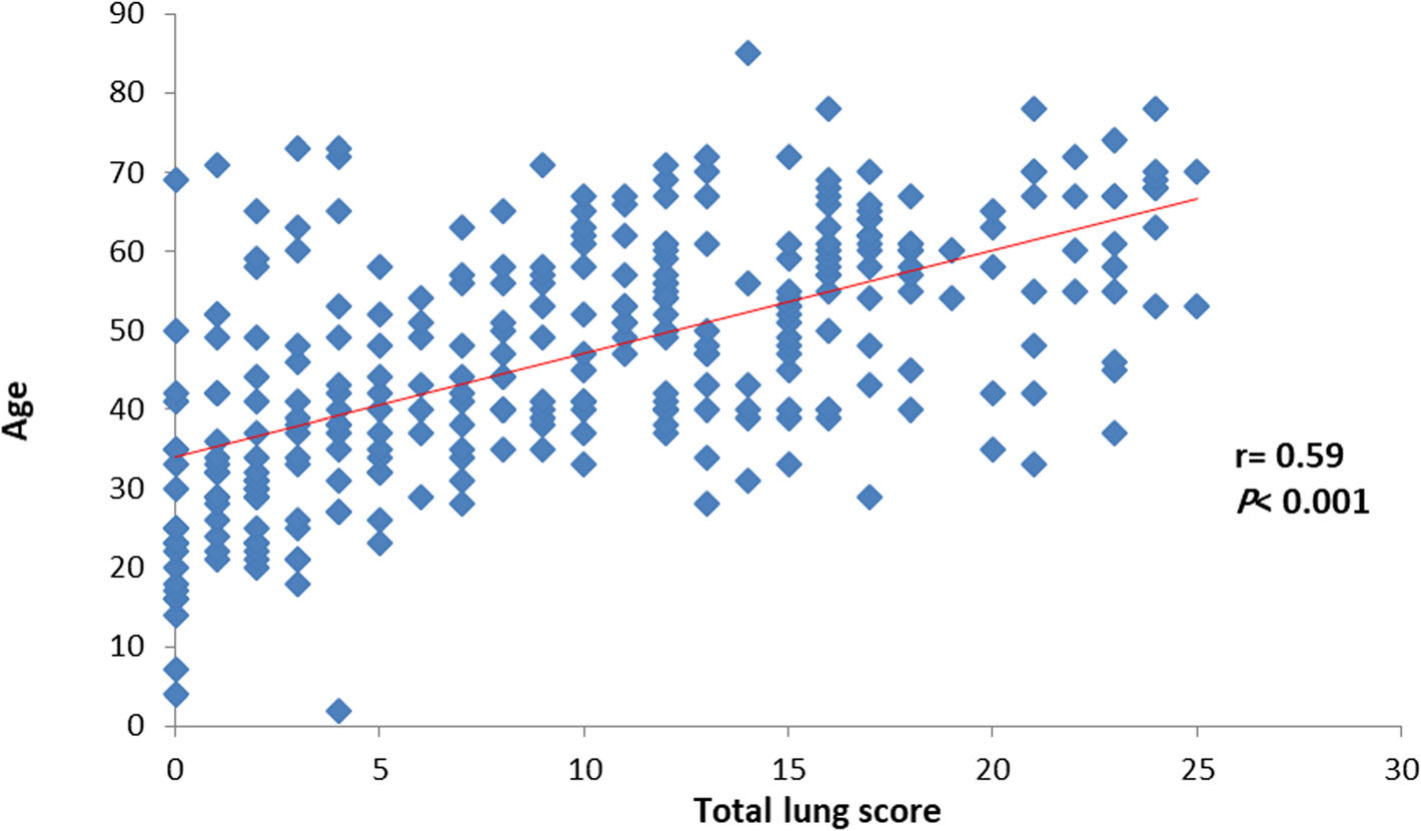
Correlation between total lung score and age in male patients

**Fig. 3:**
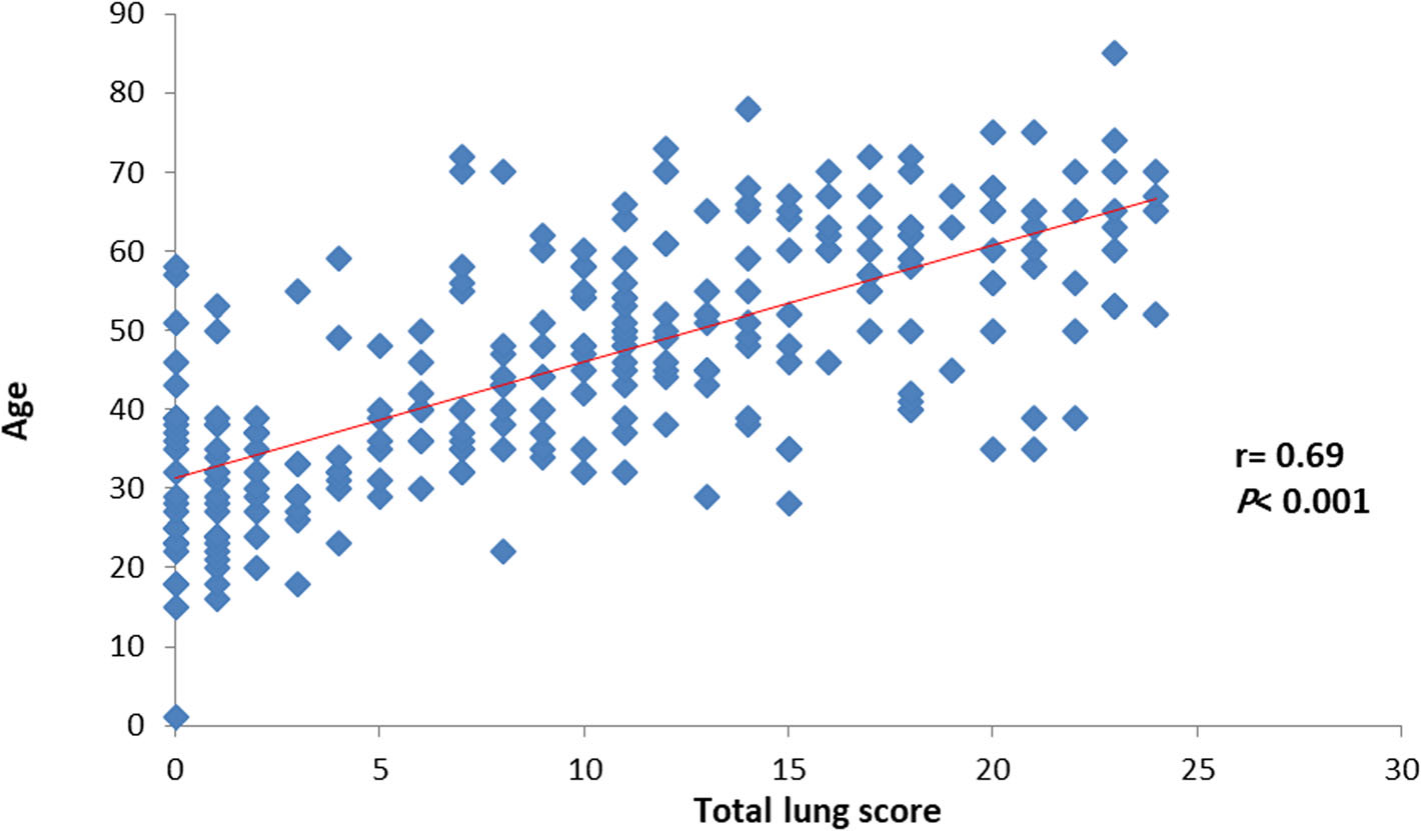
Correlation between total lung score and age in female patients

Total CT severity lung score also had a positive strong significant correlation with the age of the patient (*r =* 0.64, *P* < 0.001), and a positive strong significant correlation was observed between CT severity lung score and age in both males and females (*r =* 0.59, *P* < 0.001) and (*r =* 0.69, *P* < 0.001) respectively (Table 4), (Figs. 1, 2, and 3).

**Table 4:** Total lung score based on age group

	Total lung score		
	All patients	Males’ patients	Females’ patients
Age groups			
< 30 year	2.25 ± 1.32	2.45 ± 1.39	2.05 ± 1.27
31–39 year	6.26 ± 5.73	6.47 ± 5.76	6.09 ± 5.75
40–49 year	10.04 ± 5.19	9.84 ± 5.74	10.32 ± 4.31
50–59 year	12.48 ± 6.03	12.39 ± 5.76	12.59 ± 6.41
60–69 year	15.90 ± 5.24	15.06 ± 5.86	17.02 ± 4.07
≥ 70 year	15.96 ± 6.86	15.10 ± 7.69	17.67 ± 5.85
*P* value	< 0.001	< 0.001	< 0.001

Data expressed as mean (SD). *P* value was significant if < 0.05

Finally, it was noticed that there was a highly statistically significant difference between a grade of lung affection and age with (*P* value < 0.001), where minimal grade with a mean age (36.29 ± 12.55), while severe grade with a mean age (59.34 ± 12.01).

**Fig. 4:**
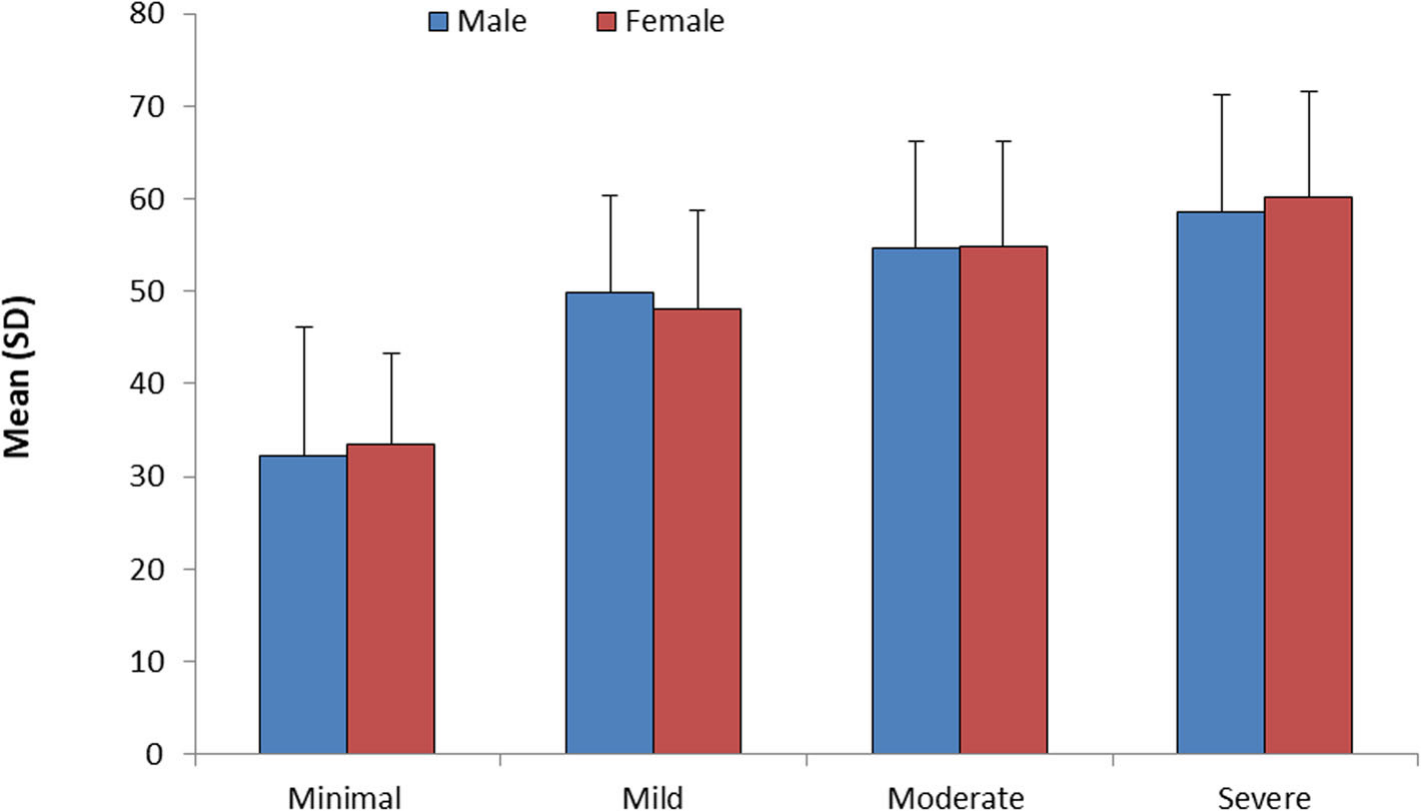
Mean age of the patients based on grades of affection based on sex

**Fig. 5:**
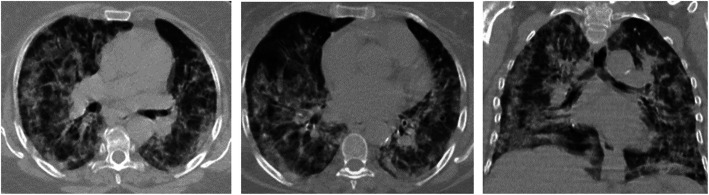
Male patient, 60 years old, was complaining of dry cough and dyspnea and was confirmed to be COVID-19 by RT-PCR. These axial and coronal non- contrast CT chest images (lung window) show typical findings with high suspicion of COVID-19 pneumonia in terms of diffuse multifocal ground-glass opacities involving both lungs with CORADS-3. Both upper and lower lobes have a score of 5 (with ≥ 75% involvement), while the middle lobe has a score of 4 with (50% to < 75 % involvement), so the total CT lung severity score is 24 with 96% of lung involvement denoting severe grade of lung affection

**Fig. 6:**
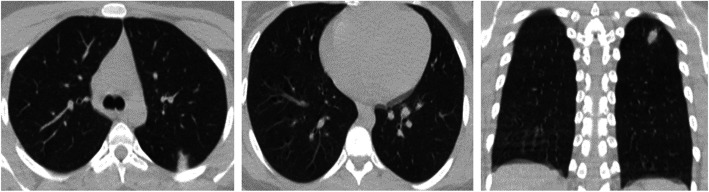
Female patient, 32 years old, was complaining of dry cough and sore throat and was confirmed to be COVID-19 by RT-PCR. These axial and coronal non- contrast CT chest images (lung window) show fairly typical findings with moderate suspicion of COVID-19 pneumonia in terms of single peripheral ground-glass opacity within the left upper lobe with CORADS-2A. Left upper lobe has score 1 (with < 5% involvement), while the right upper, middle and both lower lobes show no involvement with score 0, so total CT lung severity score is 1 with 4% lung involvement denoting minimal grade of lung affection

Also, a highly statistically significant difference was observed between the grade of lung affection and age in both males and females patients equally with (*P* value < 0.001) (Table 5), (Figs. 4, 5, and 6).

**Table 5:** Age of patients based on the grade of affection

	Age of patients		
	All patients	Males’ patients	Females’ patients
Grade of affection			
Minimal	36.29 ± 12.55	38.32 ± 13.89	33.44 ± 9.81
Mild	49 ± 10.62	49.89 ± 10.53	48.01 ± 10.71
Moderate	54.79 ± 11.36	54.75 ± 11.43	54.85 ± 11.37
Severe	59.34 ± 12.01	58.58 ± 12.60	60.13 ± 11.47
*P* value	< 0.001	< 0.001	< 0.001

Data expressed as mean (SD). *P* value was significant if < 0.05

## Discussion

On 11th February 2020, coronavirus disease (COVID-19) was declared by the World Health Organization (WHO) as the sixth global health emergency deserving the world’s attention, as it is very contagious; hence, the speed of diagnosis is crucial for rapid clinical intervention, and patient isolation [16, 17].

In this current study, 574 COVID-19 patients (311 males and 263 females) with mean age 46.70 ± 15.18 years and age range between 1 and 85 years were included. Age group C (40–49 year) was the commonest age group that was affected by COVID-19, while the least affected group was group F (≥ 70 years) by only 37 (6.4%) patients.

We found that CT features for COVID-19 patients in this study were consistent with other previous kinds of literature [16, 18], as 527 (91.8%) patients had positive CT findings. However, only 47 patients (8.2%) were presented with negative CT which is similar to the results of a previous study [19] that showed symptomatic infected patients with coronavirus, while CT chest was negative.

Bilateral lung affection was presented in 461 (80.3%) patients, while only 66 (11.5%) patients had unilateral affection and this was near matching the results of Kunwei Li et al.’s study [14], which showed bilateral involvement in (83.3%).

Structured imaging reporting system (COVID-RADS) based on typical, atypical, and fairly typical CT findings was done during this current COVID-19 global state of emergency, which is a grading system with five categories and each grade corresponds to a low, moderate, or high level of suspicion for pulmonary COVID-19 [10].

By the application of COVID-RADS in this study, we found that grade 3 which is denoting a high level of suspicion of COVID with typical CT chest findings was the most commonly presented at both sexes in all different age groups. These results can emphasize the importance of CT in screening, diagnosis, and evaluation of the severity of the disease.

Michael et al.’s study [18] introduced a method to score the severity of COVID inflammation on chest CT images that based on the summation of the degree of affection in both lung lobes to evaluate total CT severity score for overall lung involvement. And as previous literature confirmed that COVID-19 old age patients with underlying chronic diseases such as diabetes, hypertension, or cardiovascular disease are the main risk age group with high fatal outcomes [12, 13].

We used this CT severity score to quantify pulmonary involvement and correlate this severity score in different age groups and both sexes, and we found that there was a highly statistically significant correlation between age and CT severity score in both sexes with (*P* value < 0.001), as lowest CT score, was seen in patients less than 30 years; however, highest CT severity score was detected in patients above 70 years with a mean age (36.29 ± 12.55) in minimal grade and mean age (59.34 ± 12.01) in severe grade.

To the best of our knowledge, we did not find any study evaluating the correlation between CT severity score and age or sex; however, our results are matching with results of Andrea Borghes et al.’s study [1], which correlated chest X-ray severity score index in COVID-19 patients in different ages and both sexes, and showed that pulmonary involvement was significantly greater in males and females between 50 and 79 years.

## Conclusion

In conclusion, we found that age can be considered as a significant risk factor for severity of COVID-19 in both sexes. Also, we concluded that CT can be used as a significant diagnostic tool for the diagnosis of COVID-19 and evaluation of the progression and severity of the disease.

## Data Availability

The datasets used or analyzed during the current study are available from the corresponding author on reasonable request.

## Abbreviations

COVID-19: Coronavirus disease 2019
MSCT: Multislice computed tomography
GGO: Ground glass opacity
RT-PCR: Real-time polymerase chain reaction
COVID-RADS: Coronavirus imaging reporting system

## References

[1] Borghesi A (2020). Radiographic severity index in COVID-19 pneumonia: relationship to age and sex in 783 Italian patients. Radiol Med.

[2] Sun P (2020). Understanding of COVID-19 based on current evidence. J Med Virol.

[3] Huang P (2020). Use of chest CT in combination with negative RT-PCR assay for the 2019 novel coronavirus but high clinical suspicion. Radiology.

[4] Fang Y (2020). Sensitivity of Chest CT for COVID-19: Comparison to RT-PCR. Radiology.

[5] Ye G (2020). Experience of different upper respiratory tract sampling strategies for detection of COVID-19. J Hosp Infect.

[6] Xie X (2020). Chest CT for typical coronavirus disease 2019 (COVID-19) pneumonia: relationship to negative RT-PCR Testing. Radiology.

[7] Shigemura J (2020). Public responses to the novel 2019 coronavirus (2019-nCoV) in Japan: Mental health consequences and target populations. Psychiatry Clin Neurosci.

[8] Ai T (2020). Correlation of chest CT and RT-PCR testing for coronavirus disease 2019 (COVID-19) in China: a report of 1014 Cases. Radiology.

[9] Pan Y (2020). Initial CT findings and temporal changes in patients with the novel coronavirus pneumonia (2019-nCoV): a study of 63 patients in Wuhan. China Eur Radiol.

[10] Salehi S et al (2020) Coronavirus disease 2019 (COVID-19) imaging reporting and data system (COVID-RADS) and common lexicon: a proposal based on the imaging data of 37 studies. Eur Radiol 30:4930–4942. 10.1007/s00330-020-06863-0 10.1007/s00330-020-06863-0 PMC7186323 32346790

[11] Ding X (2020). Chest CT findings of COVID-19 pneumonia by the duration of symptoms. Eur J Radiol.

[12] Zhou F (2020). Clinical course and risk factors for mortality of adult inpatients with COVID-19 in Wuhan, China: a retrospective cohort study. Lancet.

[13] Shi Y (2020). Host susceptibility to severe COVID-19 and establishment of a host risk score: findings of 487 cases outside Wuhan. Crit Care.

[14] Li K (2020). CT image visual quantitative evaluation and clinical classification of coronavirus disease (COVID-19). Eur Radiol.

[15] Borghesi A, Maroldi R (2020). COVID-19 outbreak in Italy: experimental chest X-ray scoring system for quantifying and monitoring disease progression. Radiol Med.

[16] Zhu N (2020). A novel coronavirus from patients with pneumonia in China, 2019. N Engl J Med.

[17] Li Q (2020). Early transmission dynamics in Wuhan, China, of novel coronavirus-infected pneumonia. N Engl J Med.

[18] Chung M (2020). CT Imaging features of 2019 novel coronavirus (2019-nCoV). Radiology.

[19] Ling Z (2020). Asymptomatic SARS-CoV-2 infected patients with persistent negative CT findings. Eur J Radiol.

